# Metabolic pathways, genomic alterations, and post-translational modifications in pulmonary hypertension and cancer as therapeutic targets and biomarkers

**DOI:** 10.3389/fphar.2024.1490892

**Published:** 2024-11-20

**Authors:** Xiujin Zhang, Zhiqing Fu, Haijun Wang, Li Sheng

**Affiliations:** Department of Cardiology, The Second Medical Center and National Clinical Research Center for Geriatric Diseases, Chinese PLA General Hospital, Beijing, China

**Keywords:** pulmonary hypertension, metabolomics, cancer genomics, biomarkers, therapeutic targets, drug

## Abstract

**Background:**

Pulmonary hypertension (PH) can lead to right ventricular hypertrophy, significantly increasing mortality rates. This study aims to clarify PH-specific metabolites and their impact on genomic and post-translational modifications (PTMs) in cancer, evaluating DHA and EPA’s therapeutic potential to mitigate oxidative stress and inflammation.

**Methods:**

Data from 289,365 individuals were analyzed using Mendelian randomization to examine 1,400 metabolites’ causal roles in PH. Anti-inflammatory and antioxidative effects of DHA and EPA were tested in RAW 264.7 macrophages and cancer cell lines (A549, HCT116, HepG2, LNCaP). Genomic features like CNVs, DNA methylation, tumor mutation burden (TMB), and PTMs were analyzed. DHA and EPA’s effects on ROS production and cancer cell proliferation were assessed.

**Results:**

We identified 57 metabolites associated with PH risk and examined key tumor-related pathways through promoter methylation analysis. DHA and EPA significantly reduced ROS levels and inflammatory markers in macrophages, inhibited the proliferation of various cancer cell lines, and decreased nuclear translocation of SUMOylated proteins during oxidative stress and inflammatory responses. These findings suggest a potential anticancer role through the modulation of stress-related nuclear signaling, as well as a regulatory function on cellular PTMs.

**Conclusion:**

This study elucidates metabolic and PTM changes in PH and cancer, indicating DHA and EPA’s role in reducing oxidative stress and inflammation. These findings support targeting these pathways for early biomarkers and therapies, potentially improving disease management and patient outcomes.

## Background

Pulmonary hypertension (PH) is a multifactorial disorder marked by elevated pulmonary arterial pressure, often leading to right heart failure and eventual mortality (Hoendermis, 2011). Pathogenic processes in PH overlap significantly with various systemic diseases, notably cancer (Boucherat et al., 2017; Guignabert et al., 2013). Recent studies have highlighted the role of metabolic alterations in the progression of PH (Pugh and Hemnes, 2010; Maron et al., 2020; Sharma et al., 2016). Notably, PH has been linked to conditions like COVID-19, with organ-specific findings suggesting potential associations with cerebral aneurysms (Sharma et al., 2016; Finsterer, 2022). Furthermore, connections between pneumonia, stroke, and metabolic disturbances underscore the complexity of PH’s etiology, emphasizing the interplay of genetic and metabolic factors in its pathogenesis (Hassan et al., 2021). Specific metabolic pathways are implicated in PH, with studies focusing on elements such as plasma homocysteine and chronic obstructive pulmonary disease (COPD) (Hu et al., 2023; Chaudhary et al., 2019; Xu et al., 2021). The exploration of metabolic abnormalities, particularly in postmenopausal diabetic women, has potential therapeutic implications, especially considering the growing interest in nanotechnology for cancer drug delivery (Chen Y. et al., 2022).

Metabolites act as signaling molecules, influencing cellular pathways associated with PH by regulating gene expression and protein function (Milanesi et al., 2020; Han and Chandel, 2021). Research indicates that certain metabolites can affect PH through oxidative stress and inflammatory processes, often involving post-translational modifications (PTMs) such as phosphorylation, acetylation, and SUMOylation (Ebert et al., 2022; Diskin et al., 2021). The relationship between hormonal influences and vascular function provides additional insights, particularly through the role of estrogen receptors in ovarian cancer (Su et al., 2023). Tumor growth may stimulate pulmonary arteries, potentially inducing PH, while emerging genomic studies suggest an interconnectedness between pan-cancer traits and PH pathogenesis, though many underlying mechanisms remain unexplored (Chen H. et al., 2022). Recent findings on m6A RNA methylation in cancer stem cells offer novel perspectives on the genetic links between PH and cancer (Chen H. et al., 2022; Ma and Ji, 2020). PH appears to share molecular pathways with epigenetic processes involved in the epithelial-mesenchymal transition in ovarian cancer, suggesting possible commonalities in their progression (Prayudi et al., 2023).

In cancer, complex interrelations are observed among CNVs, DNA methylation, tumor mutation burden (TMB), and PTMs (Cai et al., 2019). CNVs can influence gene expression by altering gene copy numbers, potentially impacting PTM frequency (Henrichsen et al., 2009; Orozco et al., 2009). DNA methylation, through gene silencing or activation, affects PTM-encoding genes and regulates PTM levels (Qi et al., 2023; Ji et al., 2023). Increased TMB generates neoantigens that attract immune infiltration, often correlating with inflammation-related gene overexpression, potentially altering PTM patterns (Wang and Li, 2019). PTMs directly regulate protein function and, by impacting transcription factors, modulate downstream gene expression (Filtz et al., 2014). PTMs are indirectly regulated by CNVs and DNA methylation, forming a complex regulatory network that influences tumor progression and the immune microenvironment, providing crucial insights for biomarkers and targeted therapies in cancer research.

Certain metabolites function as signaling molecules under physiological and pathological conditions, potentially modulating PH-related gene expression and protein function through intracellular signaling pathways (Frezza, 2017). Studies show that specific metabolites directly affect PH by modulating oxidative stress and inflammatory responses, often involving PTM modifications of key proteins, such as phosphorylation, acetylation, and SUMOylation (Schopfer et al., 2011). As critical regulatory mechanisms for protein function, PTMs dynamically respond to cellular metabolic changes (Wang et al., 2016). For example, oxidative stress or metabolic dysregulation can alter PTMs, affecting protein stability, activity, or subcellular localization (Wang et al., 2016). This suggests that fluctuations in specific metabolites may influence PH progression by modulating PTM frequency or patterns. Metabolites might directly affect pulmonary vascular function by promoting SUMOylation, phosphorylation, or other modifications of transcription factors or structural proteins, potentially exacerbating or alleviating PH symptoms (Yao et al., 2019). Moreover, studies on the therapeutic potential of natural products in cervical cancer offer new strategies for PH treatment via metabolic and genetic pathways (Mukherjee et al., 2022). In summary, we hypothesize a close association among metabolites, PH, and PTMs, with metabolic fluctuations potentially impacting PTM-regulated protein activity and thus contributing to PH progression. This hypothesis provides a new perspective on understanding metabolite-related regulatory mechanisms in PH and establishes a foundation for studying PTMs as biomarkers and therapeutic targets in PH.

There is also an increasing need to dissect the causal relationships between metabolites and PH, alongside genomic alterations in various cancers. The current research landscape often lacks clarity regarding specific metabolite interactions with genetic changes across distinct cancer types and their implications for PH. Mendelian randomization has recently gained attention as a valuable tool in addressing these issues. By combining bioinformatics with single-cell sequencing, researchers can uncover the characteristics of immune responses within different microenvironments, providing strong data support for the development of personalized treatment plans. The advancement of these technologies has facilitated the broader application of transcriptomics, metabolomics, and proteomics in disease diagnosis and treatment (Liu et al., 2024; Chauleau and Trassin, 2024; Shi et al., 2024; Jang and Choi, 2024). This study aims to bridge existing knowledge gaps by exploring the causal relationships between metabolites and PH and examining PTM-associated genomic alterations across cancer types. By analyzing how metabolites impact genetic changes within the context of cancer, this study seeks to elucidate the pathways linking PH and cancer, providing a more comprehensive understanding of their shared mechanisms. Ultimately, our goal is to identify potential therapeutic targets and diagnostic biomarkers, advancing early detection and personalized treatment strategies. Integrating perspectives on the interactions between metabolic and genetic factors is crucial for a comprehensive understanding of complex diseases like PH.

## Materials and methods

### Integration analysis of exercise-related genes and metabolomics

The GENECARD database identifies genes associated with exercise, providing insights into their functions and relationships in various biological processes. Using the MetaboAnalyst platform, we conduct an integration study of these genes with metabolomics data.

### Genomic features of cross-over genes in pan-cancer

We use DNA methylation and copy number variation (CNV) data from The Cancer Genome Atlas (TCGA) database for our pan-cancer study. After extracting CNV data for genes that overlap in different tumor types, the genes are classified as amplifications or deletions, and the rates of each are computed to find their frequencies in distinct tumor tissues. UALCAN (http://ualcan.path.uab.edu/analysis.html) is used to analyze the promoter DNA methylation levels of overlapping genes in both normal and malignant tissues. The “Gene Visualisation” feature of the MethSurv database provides DNA methylation patterns of various malignancies. Additionally, the R package “TCGAbiolinks” is used to retrieve mutation data in the Mutation Annotation Format (MAF), and the “maftools” R package is used to compute the TMB.

### Pan-cancer GSEA enrichment analysis

Using the “limma” R package, we perform differential expression analysis between tumor and normal samples from TCGA. *P*-value criteria and log2 fold change (log2fc) are used to identify genes with substantial variations in expression. Gene Set Enrichment Analysis (GSEA) was performed using the “clusterProfiler” package in R. This analysis involved formatting the data dimensions appropriately and visualizing the results with examples to highlight significant findings.

### Tumor prognostic analysis

We assessed the predictive ability of overlapping gene expressions on patient survival outcomes using TCGA datasets, focusing on overall survival (OS). Survival analysis was conducted using the Kaplan-Meier method and log-rank test to evaluate survival across different cancer types. The “survival” and “survminer” R packages were utilized to generate survival curves. Additionally, the Cox proportional hazards model was employed to investigate the relationship between FGA and NOTCH3 expression and overall survival outcomes in pan-cancer patients, using the “forestplot” R package for visualization.

### Immune infiltration

We employ markers from the CIBERSORTx website (https://cibersortx.stanford.edu/) and the main algorithm from CIBERSORT to assess the infiltration levels of 22 immune cell types in tumor samples. This analysis is facilitated by the CIBERSORT. R script. Heat maps are used to illustrate the findings of Spearman correlation analysis, which is used to investigate the link between single-gene data and immune infiltration matrix data across pan-cancer datasets. This is a specific technique used to evaluate immune cell populations, which helps to understand the role of tumor microenvironment in cancer progression.

### Methylation analysis

Our methylation analysis specifically targets the TSS1500 region, which spans from −200 to −1,500 base pairs upstream of the transcription start site. We also focus on the TSS200 region, which is located within −200 base pairs upstream of the TSS. Additionally, we examine the first exon and the 5′untranslated region (5′UTR). Each sample’s methylation level is represented by the median value. In order to investigate the association between methylation levels and gene expression, we conduct a Spearman’s correlation analysis. The Spearman rank correlation coefficient, a non-parametric statistic, is used to assess the monotonic association between two variables, regardless of their distribution. The independent variable in this context is the methylation levels, while the dependent variable is the gene expression levels. The correlation between these two variables is evaluated by computing the Spearman rank correlation coefficient. In addition, the Wilcoxon rank-sum test is used to analyse the distribution of promoter methylation data between the tumor and normal groups.

### Gene set enrichment analysis and GSVA analysis

We conduct Gene Ontology (GO) enrichment analysis on the chosen gene sets utilising the “clusterProfiler” tool. Furthermore, the Kyoto Encyclopaedia of Genes and Genomes (KEGG) enrichment analysis is performed using the identical software package. In order to evaluate the activity of a pathway, we utilise the GSVA software and analyse the data using four different methods: zscore, gsva, ssgsea, and plage. With the exception of the zscore parameter, the scores obtained from the other techniques are transformed into unitless Z-scores using the formula (x-μ)/σ. This is done to maintain uniformity among tumor data. The Wilcoxon Rank Sum Test is used to assess statistical disparities between tumor and normal tissues. The findings are visualised by creating box plots using the ggplot2 program.

### RAW 264.7 cell line

The RAW 264.7 cell line is acquired from the Shanghai Cell Bank, which is a division of the Chinese Academy of Sciences. The cells are cultivated in RPMI-1640 medium (Gibco Invitrogen Co., San Diego, CA, United States) with the addition of 10% foetal bovine serum (Gibco BRL, Grand Island, NY, United States), 100 units/mL penicillin, and 0.1 mg/mL streptomycin. The culture conditions are upheld at a temperature of 37°C, with a humidity level of 95% and a CO_2_ concentration of 5%. Docosahexaenoic acid (DHA) is dissolved in dimethyl sulfoxide (DMSO) to form a 1 millimolar (mM) concentrated solution, which is then kept at a temperature of −20°C. During the experiment, the stock solution is mixed with DMSO to provide a final concentration of 2 μM DHA and EPA for the RAW 264.7 cells. These cells are then subjected to a 48-h treatment. The DHA (D2534, purity >99%) and a standard for 37 fatty acids are obtained from Sigma (St. Louis, MO, United States), whereas eicosapentaenoic acid (EPA) is acquired from Sigma-Aldrich (St. Louis, MO, United States).

### Cell culture and colony formation assay

The following cell lines—LNCaP (prostate cancer), HepG2 (liver cancer), A549 (lung adenocarcinoma), and HCT116 (colorectal cancer)—were cultured under standard conditions. Each cell line was maintained in Dulbecco’s Modified Eagle Medium (DMEM) supplemented with 10% fetal bovine serum (FBS) and 1% penicillin-streptomycin. Cells were incubated at 37°C in a humidified atmosphere containing 5% CO₂. The culture medium was replaced every 2–3 days, and cells were subcultured upon reaching 80%–90% confluence using 0.25% trypsin-EDTA for detachment. All cell lines were authenticated prior to experiments to ensure reliability and reproducibility of the results. For the colony formation assay, cells were seeded into 6-well plates at a density of 500–1,000 cells per well and allowed to adhere overnight. The cells were then cultured in DMEM supplemented with 10% FBS at 37°C in a humidified incubator with 5% CO₂. The medium was refreshed every 3–4 days. After 10–14 days, when visible colonies had formed, the medium was removed, and the cells were gently washed twice with phosphate-buffered saline (PBS). The colonies were fixed with 4% paraformaldehyde for 15 min at room temperature and subsequently stained with 0.5% crystal violet solution for 20 min. After washing away the residual stain with tap water, the plate was air-dried. After 7–10 days, colonies containing more than 50 cells were counted under a microscope, and the colony formation efficiency was calculated as the number of colonies divided by the number of cells seeded.

### Inhibition of reactive oxygen species (ROS) production

The RAW 264.7 cells were diluted to a concentration of 250,000 cells/mL. Luminol, a light-enhancing compound, and zymosan, which stimulates the production of ROS, were added to each well. The resulting chemiluminescent emission from the formation of ROS was then measured.

### Immunofluorescence detection

Immunofluorescence detection was performed as previously described (Im et al., 2019; Chen et al., 2024a). Cells were initially fixed with a 4% paraformaldehyde (PFA) solution for 15 min, followed by blocking for 1 h. The samples were then incubated overnight with the primary antibody at 4°C. On the second day, the samples were brought to room temperature (25°C) and stained with a fluorescently labeled secondary antibody for 1 h. After washing with PBS, a coverslip was mounted using an anti-fade mounting solution containing DAPI from Abcam. Fluorescence microscopy was then used to capture images.

### Statistical analysis

A *P*-value of <0.05 was considered statistically significant, ensuring the reliability and accuracy of our findings regarding metabolites and pulmonary hypertension. Mean ± standard errors are reported for the data.

## Results

### Causal relationship between metabolites and the risk of pulmonary hypertension

**FIGURE 1:**
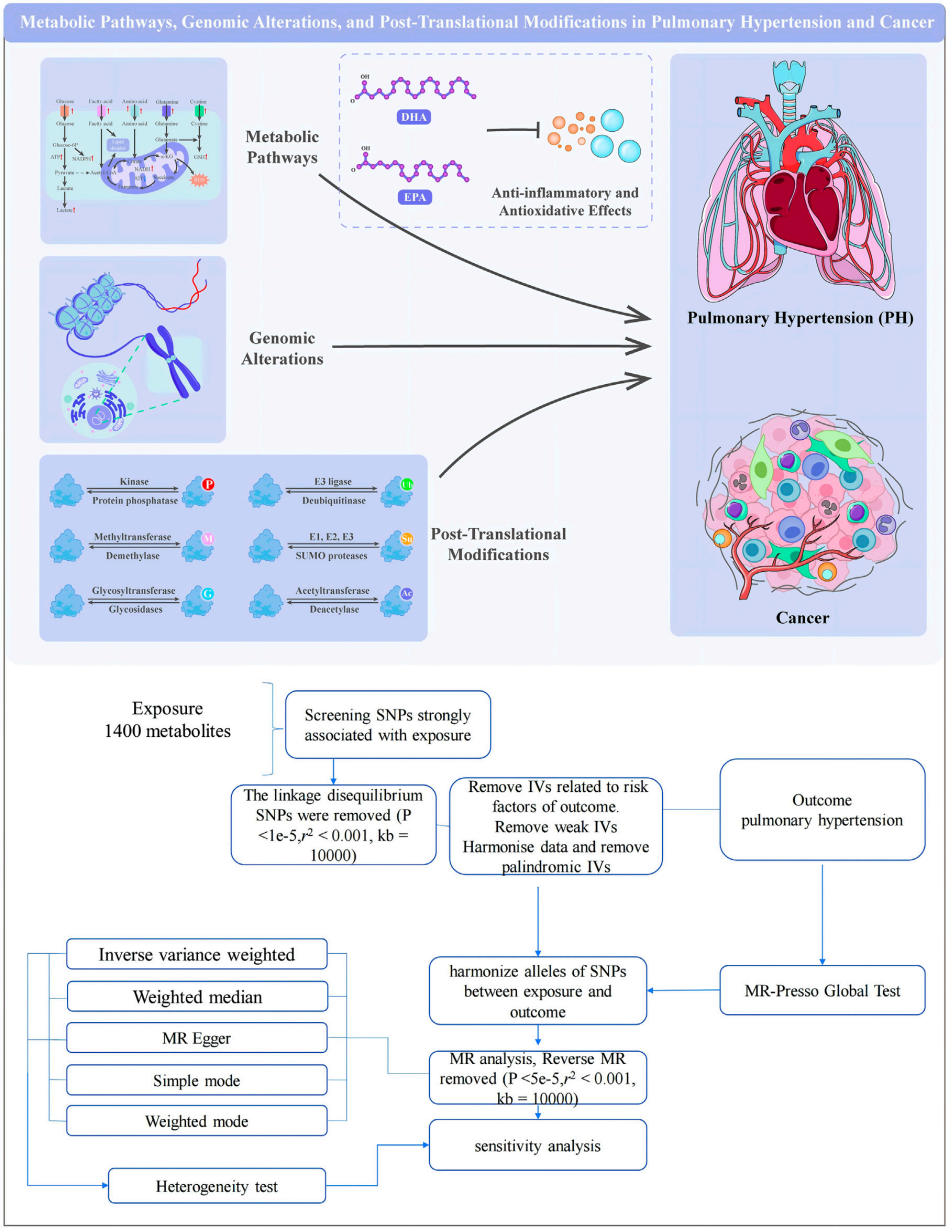
Flowchart of MR Analysis for Pulmonary Hypertension. The flowchart above depicts the systematic approach employed in our mediation analysis to investigate the correlation between 1,400 metabolites and the incidence of pulmonary hypertension. The process begins with the introduction of the 1,400 metabolites, followed by screening for single nucleotide polymorphisms (SNPs) strongly associated with them. SNPs in linkage disequilibrium, characterized by an r^2^ value greater than 0.0001 and a *p*-value less than 1e-5, are excluded if they are within 10,000 base pairs of each other. Excluded from consideration are instrumental variables (IVs) that are associated with possible confounders, and instrumental variables that lack sufficient power are also avoided. The data are standardised, and IVs that are palindromic are removed to ensure a reliable introduction of alleles between the exposure and result SNPs. The specific result being studied is pulmonary hypertension. Single nucleotide polymorphisms (SNPs) are standardised or made consistent across the initial and final datasets. Reverse MR is conducted, and the analysis of MR proceeds with the remaining significant independent variables that have a *p*-value less than 5e-5, an r-squared value less than 0.001, and a coefficient of 10,000. Multiple MR techniques are employed to evaluate causation, such as IVW, Weighted Median, MR Egger, Simple Mode, and Weighted Mode. A heterogeneity test is performed to assess the uniformity of the outcomes across various tactics. The MR-PRESSO Global Test is employed to identify and address horizontal pleiotropy. Sensitivity analysis is conducted to verify the reliability and stability of the results. This comprehensive method ensures that the identified connections between metabolites and pulmonary hypertension are strong and less likely to be confused by pleiotropy or other predispositions.

To address this inquiry, we utilized a two-sample MR methodology to evaluate the potential causal relationship between metabolites and the risk of PH. The intercept test conducted using MR Egger indicated no signs of horizontal pleiotropy (*p* > 0.05 for all instrumental variables), thereby reinforcing the reliability of our results. Additionally, the outcomes of the leave-one-out sensitivity analysis, employing a jackknife approach, are presented in Figure 1. Our analysis identified 57 metabolites with a significant causal association with the development of PH, using a significance threshold of 0.01 (Supplementary Figure S1). Among these, metabolites such as Glucuronide of piperine (*P* = 0.030, OR = 1.316, 95% CI = 1.026–1.689), N-lactoyl valine (*P* = 0.034, OR = 2.029, 95% CI = 1.052–3.911), and N-stearoyl-sphingadienine (d18:2/18:0) (*P* = 0.007, OR = 1.570, 95% CI = 1.126–2.189) were found to be positively associated with an increased risk of PH. Conversely, metabolites such as Cerotoylcarnitine (C26) (*P* = 0.027, OR = 0.688, 95% CI = 0.493–0.959), Docosatrienoate (22:3n6) (*P* = 0.041, OR = 0.642, 95% CI = 0.420–0.982), and 5-dodecenoylcarnitine (C12:1) (*P* = 0.036, OR = 0.545, 95% CI = 0.309–0.962) exhibited a protective effect against PH, showing negative correlations. Further reverse MR analysis assessed the impact of PH on these metabolites. The results suggested a negative correlation between PH and S-methylcysteine levels (*P* = 0.045, OR = 1.014, 95% CI = 1.000–1.028). The MR Forest Plot (Supplementary Figure S1) visually represents the odds ratios (ORs) and 95% confidence intervals (CIs) for each metabolite, with data analyzed across different MR techniques. The metabolites examined include a broad spectrum of biological compounds, such as amino acids, lipids, and carbohydrates. In the forest plot, horizontal lines representing the ORs and CIs indicate significant associations when they do not cross the null value (OR = 1). To ensure robust causal inference, we employed multiple MR methodologies, including Inverse-Variance Weighted (IVW), MR Egger, weighted median, weighted mode, and simple mode approaches, using genetic predictors derived from genome-wide association studies (GWAS). The IVW method combined the effect estimates of genetic instruments, MR Egger addressed directional pleiotropy, and the weighted median provided reliable estimates even when up to 50% of the data came from invalid instruments. These diverse approaches strengthen the robustness and validity of our findings. This comprehensive analysis underscores significant causal relationships between specific metabolites and the risk of PH, identifying potential therapeutic targets and biomarkers for disease prevention and management.

### Identification of metabolic pathways and exercise-related genes associated with pulmonary hypertension

**FIGURE 2:**
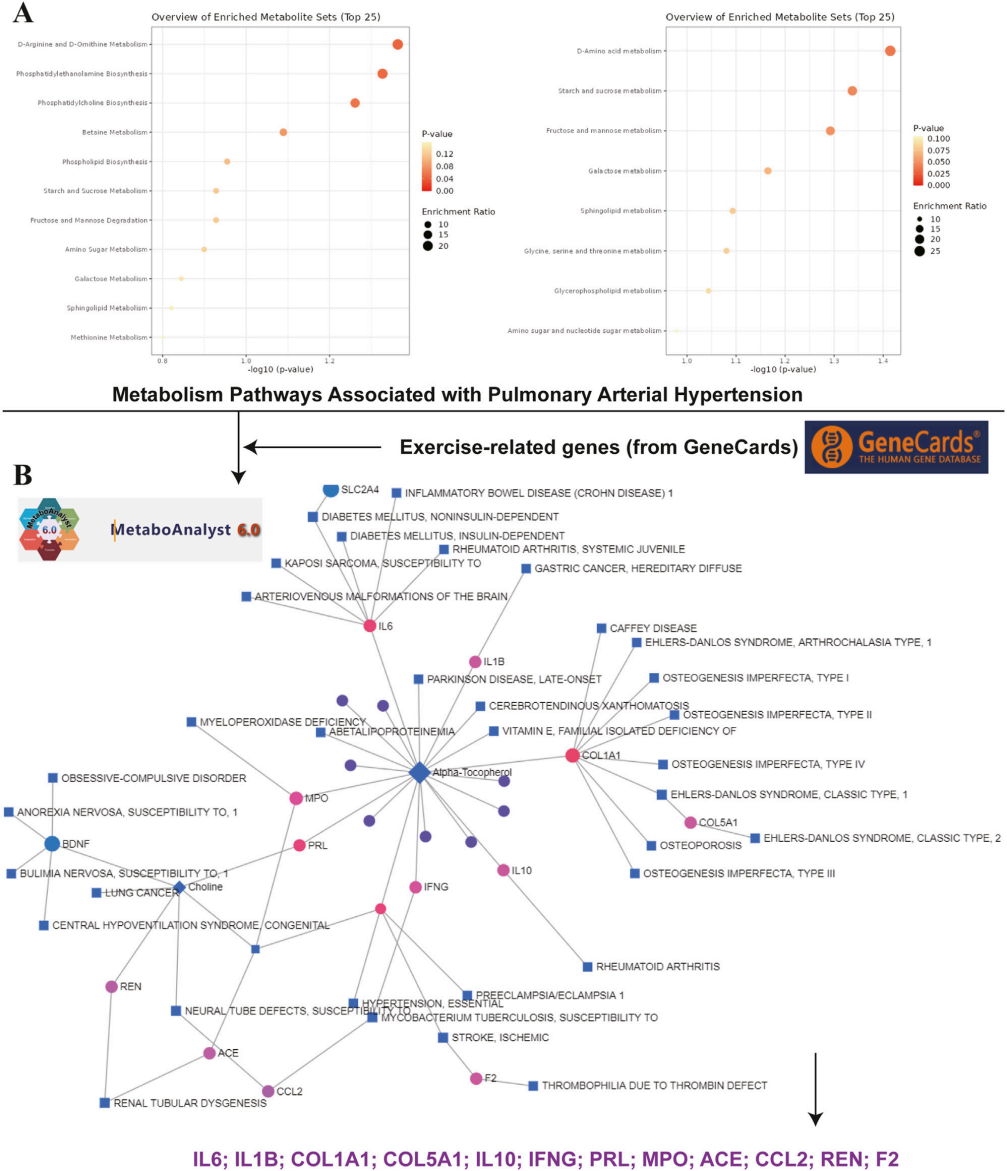
Metabolic Pathways and Exercise-Related Genes Related to Pulmonary Hypertension. **(A)** This section presents the top 25 metabolic pathways significantly associated with pulmonary hypertension (PH). On the left, pathways are displayed based on their *p*-values, while the right side features those with high enrichment ratios. Each dot represents a distinct pathway, colored by *p*-value (with red indicating more significant values) and sized according to the enrichment ratio. Data analysis was conducted using the MetaboAnalyst 6.0 platform, which provides comprehensive metabolomics data analysis and visualization. The pathways were identified from metabolomics data collected from patients with PH. **(B)** This well-structured network illustrates the associations between exercise-related genes and various diseases or disorders. Using the GeneCards database, genes such as IL6, IL1β, COL1A1, COL5A1, IL10, IFNG, PRL, MPO, ACE, CCL2, REN, and F2 were identified, along with detailed gene information. In the network, nodes represent the genes, while edges indicate their associations with diseases. These genes are linked to a range of conditions, including inflammatory bowel disease, diabetes mellitus, rheumatoid arthritis, and Parkinson’s disease, among others. The colors and sizes of the nodes reflect the strength and nature of the links, as well as their importance. This network aims to enhance understanding of the broader impact of these genes on various physiological and pathophysiological states, with particular emphasis on pulmonary hypertension and exercise physiology.

The analysis of metabolic pathways and exercise-related genes in the context of PH provided significant insights into potential therapeutic targets. Figure 2A depicts the top 25 enriched metabolic pathways identified from metabolomics data of PH patients, analyzed using the MetaboAnalyst 6.0 platform. The pathways are ranked by *p*-value (left panel) and enrichment ratio (right panel). Pathways such as D-Arginine and D-Ornithine metabolism, starch and sucrose metabolism, and galactose metabolism exhibited significant associations with PH. The size and color of the dots represent the enrichment ratio and statistical significance, respectively, with higher ratios and more significant *p*-values highlighted in red. These findings suggest that disturbances in amino acid and carbohydrate metabolism play crucial roles in the pathophysiology of PH, potentially offering novel avenues for metabolic intervention. In Figure 2B, a network of exercise-related genes and their associations with various diseases was constructed using data from the GeneCards database. The network includes genes such as IL6, IL1B, COL1A1, COL5A1, IL10, IFNG, PRL, MPO, ACE, CCL2, REN, and F2, which are linked to a wide array of diseases, including inflammatory bowel disease, diabetes mellitus, rheumatoid arthritis, and Parkinson’s disease. These genes are known to play critical roles in inflammation, immune regulation, and metabolic processes. The network analysis underscores the relevance of these genes in the physiological response to exercise and their potential role in modulating the immune and metabolic dysregulation observed in PH. The identification of key metabolic pathways and gene-disease associations provides a foundation for future research aimed at developing exercise-based therapeutic strategies, which could modulate these metabolic and immune processes to improve outcomes for PH patients.

### Prognostic significance of overlapping gene expression in various cancers

**FIGURE 3:**
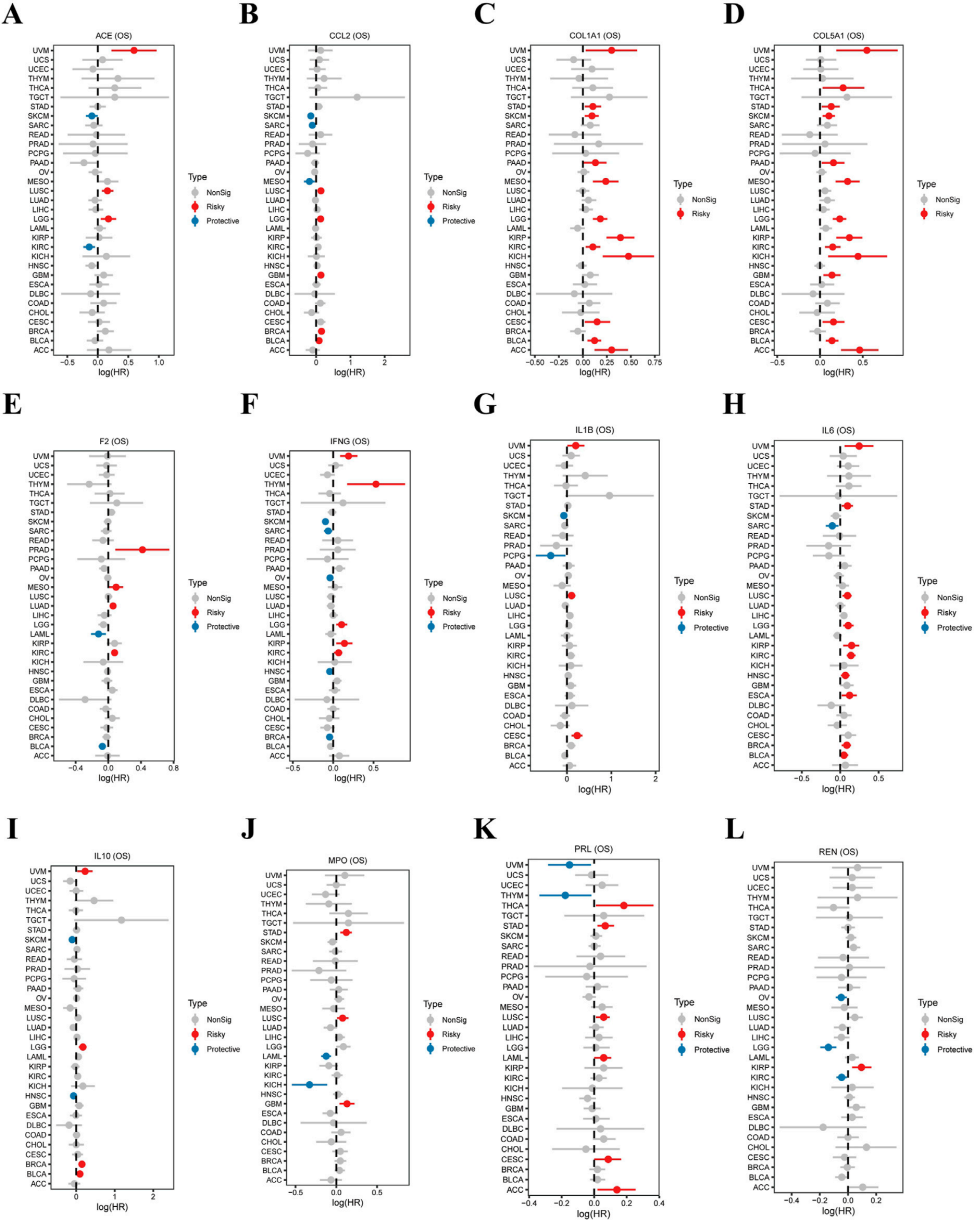
Expression of Overlapping Genes and Their Correlation with Tumor Prognosis. This figure presents forest plots illustrating the hazard ratios (HRs) and 95% confidence intervals (CIs) for 12 overlapping genes (ACE, CCL2, COL1A1, COL5A1, F2, IFNG, IL1β, IL6, IL10, MPO, PRL, and REN) across various cancers. The plots depict the relationship between the expression patterns of these genes and OS, highlighting their prognostic significance in cancer outcomes. Each plot presents the gene expression data in multiple cancers, highlighting both protective and risky associations with survival outcomes. **(A)** ACE (OS): High ACE expression was protective in kidney renal clear cell carcinoma (KIRC) and mesothelioma (MESO), but risky in uterine carcinosarcoma (UCS). **(B)** CCL2 (OS): Elevated CCL2 expression was linked to higher risk in kidney renal papillary cell carcinoma (KIRP) and low-grade glioma (LGG). **(C)** COL1A1 (OS): COL1A1 expression demonstrated risky trends in breast invasive carcinoma (BRCA), head and neck squamous cell carcinoma (HNSC), and stomach adenocarcinoma (STAD), while showing protective effects in kidney chromophobe (KICH). **(D)** COL5A1 (OS): High COL5A1 expression was associated with increased risk in liver hepatocellular carcinoma (LIHC) and lung adenocarcinoma (LUAD), but protective in KIRC. **(E)** F2 (OS): F2 expression was predominantly protective in KIRC and bladder urothelial carcinoma (BLCA), but risky in sarcoma (SARC) and LGG. **(F)** IFNG (OS): IFNG expression showed protective trends in esophageal carcinoma (ESCA) and KIRC, but risky in BRCA. **(G)** IL1β (OS): IL1B expression was protective in KIRC and BRCA, but associated with increased risk in stomach adenocarcinoma (STAD) and skin cutaneous melanoma (SKCM). **(H)** IL6 (OS): Elevated IL6 expression was linked to risky prognosis in lung squamous cell carcinoma (LUSC) and BLCA, but protective in KIRC. **(I)** IL10 (OS): IL10 expression was associated with increased risk in KIRP and pancreatic adenocarcinoma (PAAD), while showing protective effects in LUSC. **(J)** MPO (OS): MPO expression demonstrated risky trends in liver hepatocellular carcinoma (LIHC) and LUSC, while being protective in BLCA. **(K)** PRL (OS): High PRL expression posed a risk in colon adenocarcinoma (COAD) and BRCA, but was protective in KIRC. **(L)** REN (OS): REN expression was protective in KIRC and kidney renal papillary cell carcinoma (KIRP), but risky in low-grade glioma (LGG) and UCS.

This study investigates the prognostic relevance of 12 overlapping genes (ACE, CCL2, COL1A1, COL5A1, F2, IFNG, IL1B, IL6, IL10, MPO, PRL, and REN) across different cancer types by analyzing their correlation with overall survival (OS). The hazard ratios (HRs) and 95% confidence intervals (CIs) for each gene’s expression were calculated, revealing important trends in cancer prognosis. As illustrated in Figure 3A, ACE expression is protective against kidney renal clear cell carcinoma (KIRC) and mesothelioma (MESO) but may present risks in uterine carcinosarcoma (UCS). Elevated ACE levels confer significant advantages for certain cancers while being detrimental to others. Regarding CCL2 (Figure 3B), increased expression correlates with higher risk in kidney renal papillary cell carcinoma (KIRP) and low-grade glioma (LGG), indicating its role as a negative prognostic marker for these malignancies. COL1A1 expression is associated with risk in various cancers (Figure 3C), including breast invasive carcinoma (BRCA), head and neck squamous cell carcinoma (HNSC), and stomach adenocarcinoma (STAD), whereas it provides protective effects in kidney chromophobe (KICH), highlighting its complex role depending on cancer type. For COL5A1 (Figure 3D), high expression is linked to increased risk in liver hepatocellular carcinoma (LIHC) and lung adenocarcinoma (LUAD) but offers protective benefits for KIRC, demonstrating its dual nature in cancer prognosis. The F2 gene (Figure 3E) predominantly exhibits protective effects across various cancers, although it poses risks in KIRC and bladder urothelial carcinoma (BLCA). Similarly, cancers like sarcoma (SARC) and LGG present adverse outcomes, reflecting the gene’s varied impact on different tumor types. In terms of IFNG (Figure 3F), sensitivity to expression levels is evident, as it shows protective trends in esophageal carcinoma (ESCA) and KIRC, yet poses risks in BRCA. A similar pattern is observed with IL1B (Figure 3G), which has crucial protective roles in KIRC and BRCA while being associated with significantly higher risks for stomach adenocarcinoma (STAD) and skin cutaneous melanoma (SKCM), despite its complex involvement in inflammatory pathways within tumors. For IL6 (Figure 3H), high expression indicates a poor prognosis in lung squamous cell carcinoma (LUSC) and BLCA, while demonstrating protective effects for KIRC, illustrating the dual aspects of cancer progression. IL10 (Figure 3I) correlates with increased risk in KIRP and pancreatic adenocarcinoma (PAAD), yet displays protective tendencies in LUSC, emphasizing its variable role across different tumor microenvironments. MPO (Figure 3J) becomes concerning, as elevated expression is associated with LIHC and LUSC, while providing protective benefits in BLCA, indicating its differing impacts based on tumor type. Lastly, PRL (Figure 3K) shows high expression linked to risks in colon adenocarcinoma (COAD) and BRCA, yet has favorable effects for KIRC, showcasing the diverse roles this gene plays in tumor biology. Finally, REN (Figure 3L) exhibited protective trends in cancers such as KIRC and kidney renal papillary cell carcinoma (KIRP), while being associated with increased risk in LGG and UCS, highlighting its potential as a prognostic marker.

### Analysis of copy number variation, methylation, and TMB of overlapping genes in pan-cancer

**FIGURE 4:**
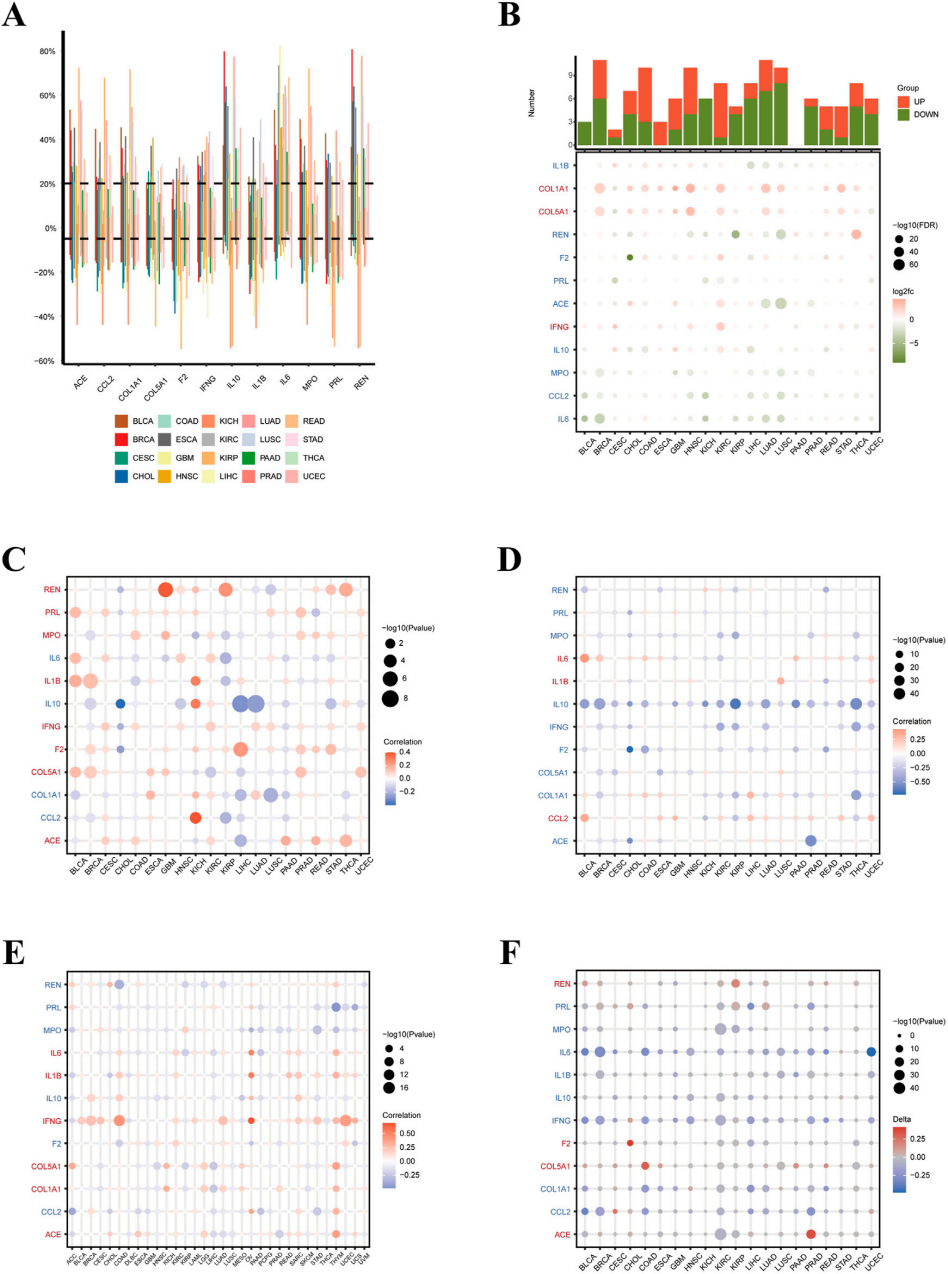
Analysis of Copy Number Variation, Methylation, and TMB of Overlapping Genes in Pan-Cancer. **(A)** Distribution of copy number variation (CNV) rates among overlapping genes across 20 different cancer types. The graph shows the variation in CNV for each gene across selected cancer types, with distinct colors representing different cancers. **(B)** Differential expression analysis of overlapping genes across multiple tumors. The top bar chart represents the number of genes that are upregulated (red) or downregulated (green) in each cancer type. Below, the dot plot shows the expression levels of these genes, with the size of each dot reflecting the significance of differential expression (-log10(FDR)) and the color indicating the log2 fold change. **(C)** Association of gene dosage with expression changes in different cancers. The dot plot displays the correlation coefficients (color-coded) between CNV and gene expression levels. **(D)** Relationship between promoter methylation and gene expression in different cancers. This plot illustrates the correlation coefficients (color-coded) for the effect of promoter methylation changes on gene expression. **(E)** Association between TMB and gene expression in distinct malignancies. The dot plot presents the correlation coefficients (color-coded) for the relationship between TMB and gene expression levels, with the dot size indicating the significance (-log10 (*p*-value)) of these associations. This analysis helps to identify genes whose expression is influenced by the overall mutation load in tumors. **(F)** δ-values of promoter methylation in tumors versus normal tissues for overlapping genes across different cancers. This graphic displays the δ-values (difference in methylation levels) between tumors and normal tissues, with the size of the dots indicating the significance (-log10 (*p*-value)).

Next, we explored CNV, methylation, and TMB analysis of pan cancer overlapping genes to clarify their functional characteristics. Figure 4A shows the distribution of CNV rates among the overlapping genes across 20 different cancer types. Each bar in the graph represents the variation in CNV for individual genes across these cancers, with distinct colors indicating different cancer types. For instance, COL1A1 and COL5A1 showed significant CNV gains in cancers like BRCA and STAD, while ACE and IFNG exhibited frequent CNV losses in KIRC and LUAD. Figure 4B presents the differential expression analysis of these overlapping genes across various tumors. Notably, IL6 and IL10 were significantly upregulated in cancers such as PAAD and LIHC, suggesting their potential roles in tumor progression and inflammation. Figure 4C explores the correlation between CNV and gene expression in various malignancies. This figure reveals that genes like COL1A1 and COL5A1 display strong positive correlations between CNV and expression levels in cancers such as BRCA and STAD, indicating that CNV gains directly enhance the expression of these genes. Conversely, negative correlations were observed for IFNG in LUAD, suggesting that CNV losses are associated with reduced gene expression in certain contexts. Figure 4D delves into the relationship between promoter methylation and gene expression across different cancers. The data show that hypermethylation of genes like ACE and F2 is associated with decreased expression in tumors such as LUAD and KIRC, underscoring the role of epigenetic silencing in these cancers. On the other hand, IL1B and IL6 exhibited hypomethylation coupled with increased expression in cancers like colorectal adenocarcinoma (COAD) and BRCA, indicating that promoter demethylation may activate oncogenic pathways in these tumors. Figure 4E highlights the association between TMB and gene expression across various cancer types. This analysis reveals that genes such as IL6 and MPO show significant positive correlations with TMB in high-mutation cancers like LUSC and esophageal carcinoma (ESCA), suggesting that higher mutation loads might drive the overexpression of these inflammatory mediators. Conversely, genes like REN and PRL show a negative correlation with TMB in cancers like KIRC, implying that these genes might be suppressed in tumors with a high mutation burden. Figure 4F illustrates the δ-values of promoter methylation in tumors versus normal tissues for the overlapping genes across different cancers. The data indicate that COL5A1 and MPO exhibit significant hypermethylation in tumor tissues compared to normal tissues in cancers like STAD and BRCA, which is associated with their reduced expression. Conversely, IL10 and IFNG exhibit hypomethylation in pancreatic adenocarcinoma (PAAD) and lung squamous cell carcinoma (LUSC) tumors, which correlates positively with elevated mRNA levels. This suggests their potential role in fostering a pro-tumorigenic microenvironment. The GSEA was performed on a diverse set of cancer types, including cervical squamous cell carcinoma and endocervical adenocarcinoma (CESC), glioblastoma multiforme (GBM), head and neck squamous cell carcinoma (HNSC), kidney renal clear cell carcinoma (KIRC), acute myeloid leukemia (LAML), liver hepatocellular carcinoma (LIHC), lung adenocarcinoma (LUAD), lung squamous cell carcinoma (LUSC), ovarian serous cystadenocarcinoma (OV), pancreatic adenocarcinoma (PAAD), prostate adenocarcinoma (PRAD), rectum adenocarcinoma (READ), skin cutaneous melanoma (SKCM), stomach adenocarcinoma (STAD), testicular germ cell tumors (TGCT), and uveal melanoma (UVM) (Supplementary Figure S2). A comprehensive analysis of thousands of pathways was conducted, encompassing critical cellular processes such as the canonical xenobiotic metabolism system, WNT beta-catenin signaling, unfolded protein response, and epithelial to mesenchymal transition (EMT), among others.

### Correlation of overlapping gene expression with immune cell infiltration and minor allele frequency across cancers

**FIGURE 5:**
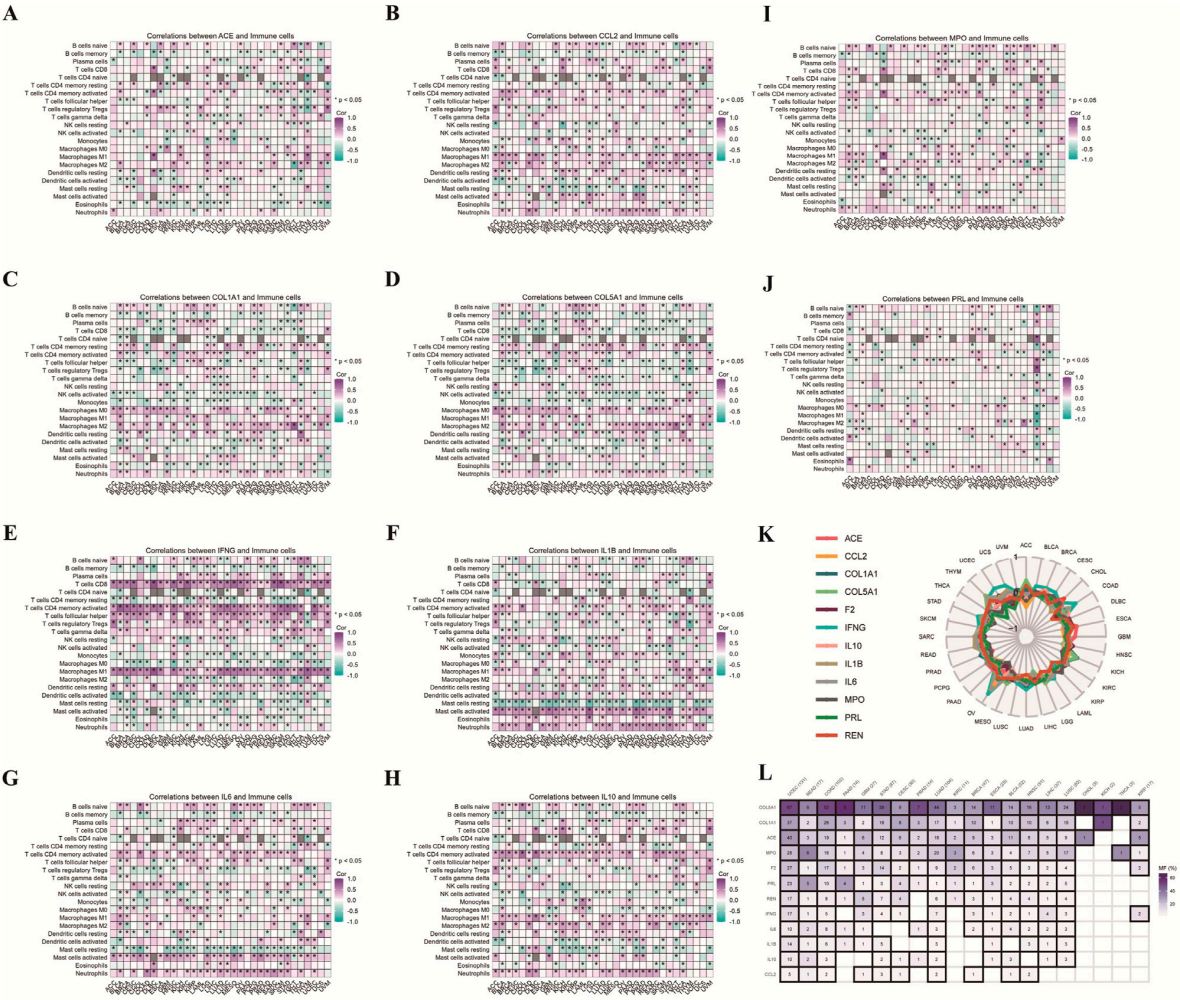
Correlation Analysis of Overlapping Genes with Immune Cell Infiltration. **(A–J)** Heatmaps showing correlations between the expression of key genes (ACE, CCL2, COL1A1, COL5A1, F2, IFNG, IL1β, IL6, IL10, MPO, PRL, and REN) and the infiltration of various immune cell types across different tumor types. Each subfigure **(A–J)** illustrates the correlation between the expression of a single gene and the abundance of multiple immune cell types, including B cells, T cells (CD8^+^ and CD4^+^ subsets), NK cells, macrophages, dendritic cells, and mast cells. The color intensity in the heatmaps corresponds to the correlation coefficient (r), where purple indicates positive correlations and green indicates negative correlations. The significance of the correlations is denoted by the size of the squares, with larger squares indicating higher statistical significance (*p* < 0.05). **(K)** Radial plot representing the expression patterns of key overlapping genes (ACE, CCL2, COL1A1, COL5A1, F2, IFNG, IL1β, IL6, IL10, MPO, PRL, and REN) across various cancer types in relation to TMB. The circumference of the plot represents different cancer types, while the colored lines indicate the expression patterns of each gene in relation to TMB, with data points standardized for comparison. Pearson correlation coefficients were used to assess the strength of these relationships. **(L)** Heatmap depicting the MAF of the overlapping genes across different cancer types. The intensity of the color in each cell represents the frequency of minor alleles, with deeper hues indicating higher frequency. Each cell corresponds to a specific gene-cancer type combination, derived from extensive cancer genomics studies. The statistical significance of the MAF data was determined using chi-square tests.

In this study, we conducted a comprehensive pan-cancer analysis of the relationship between the expression levels of key overlapping genes across various cancer types and immune cell infiltration, TMB, and MAF, highlighting the crucial roles of these overlapping genes. Using heatmaps, radial plots, and MAF heatmaps generated by CIBERSORT, we systematically evaluated these relationships. The heatmaps presented in Figures 5A–J show significant correlations between the expression levels of key genes (ACE, CCL2, COL1A1, COL5A1, F2, IFNG, IL1B, IL6, IL10, MPO, PRL, and REN) and immune cell infiltration levels, including B cells, CD8^+^ T cells, regulatory T cells (Tregs), natural killer (NK) cells, macrophages, immature dendritic cells (iDC), activated dendritic cells (aDC), and mast cells. Our findings provide strong evidence of the association between gene expression and immune cell infiltration from a pan-cancer perspective. To illustrate the expression patterns of these TMB-associated overlapping genes across cancer types, radial plot analysis revealed a bimodal distribution of TMB-related gene expression levels, suggesting their potential role as regulatory factors in cancer mutation processes (Figure 5K). Additionally, the heatmap in Figure 5L shows the MAF of these overlapping genes across different cancer types, with observed MAF differences potentially reflecting the heterogeneity of genetic alterations and selective pressures encountered in tumorigenesis. Overall, this study reveals critical insights into the genetic and immunological characteristics of cancer, suggests potential biomarkers for cancer detection and prognosis, and highlights molecular mechanisms that may drive tumor progression.

### Pathway analysis in various tumors

**FIGURE 6:**
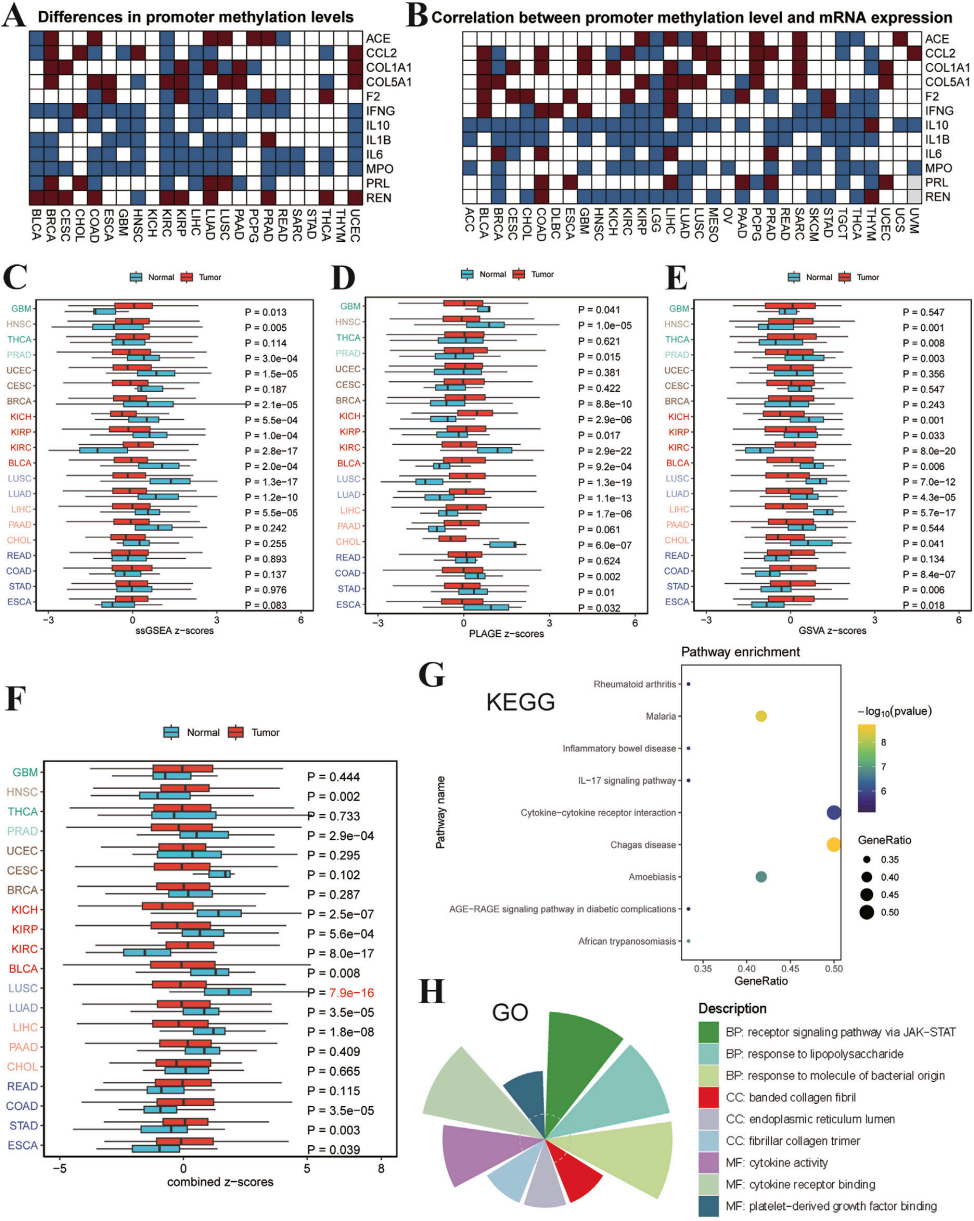
Comprehensive Analysis of Promoter Methylation, Gene Expression Correlation, Pathway Activity, and Enrichment Analysis in Various Tumors. **(A)** Heatmap showing differences in promoter methylation levels across multiple cancers. Each row represents a specific gene, while each column indicates a type of cancer. Red represents higher promoter methylation levels in tumor tissues compared to normal tissues, while blue indicates lower promoter methylation levels in tumor tissues. **(B)** Heatmap illustrating the correlation between promoter methylation levels and mRNA expression in distinct cancers. Each row represents a gene, and each column indicates a type of cancer. Red indicates a positive correlation between promoter methylation levels and gene expression, while blue indicates a negative correlation, highlighting the complex relationship between methylation and gene expression. **(C–F)** Boxplots depicting pathway activity Z-scores in tumor tissues versus normal tissues across various cancers. The vertical axis lists different types of cancer, while the horizontal axis represents pathway activity Z-scores. Wilcoxon Rank Sum Tests were used to assess statistical significance when comparing tumor and normal tissues. **(G)** Scatter plot displaying KEGG pathway enrichment analysis. The color gradient from yellow to blue represents the level of significance, with yellow being more significant. The size of each dot corresponds to the fraction of enriched genes within each pathway, highlighting the degree of enrichment and identifying critical pathways involved in tumorigenesis. **(H)** Radial plot illustrating Gene Ontology (GO) enrichment analysis. The radius of each sector represents the negative log10 of the adjusted *p*-value, with larger sectors indicating higher levels of enrichment. The gray circle marks the position where the adjusted *p*-value equals 0.05. This plot emphasizes the most significantly enriched GO terms, providing insights into the molecular functions and biological processes that are critically altered in cancer.

An integrated analysis of promoter methylation and its correlation with gene expression, alongside pathway activity and enrichment, can yield valuable insights into tumorigenesis across different cancers. Heatmaps presented in Figure 6A illustrate the varying levels of promoter methylation among multiple cancer types, with red indicating significantly higher methylation and blue indicating substantially lower methylation when comparing tumor tissues to normal tissues. In Figure 6B, we plot promoter methylation levels against mRNA expression, where red represents a positive correlation and blue indicates a negative correlation, highlighting the complex interplay between methylation and gene expression. Subsequent boxplots (Figures 6C–F) present pathway activity Z-scores in tumor tissues versus normal tissues across different cancers, with the Wilcoxon Rank Sum Test employed to assess statistical significance. These plots reveal significant differences in pathway activity between tumor and normal tissues, with distinct patterns observed in specific cancer types. Figure 6G offers a scatter plot of KEGG pathway enrichment analysis, where the color gradient from yellow to blue represents the level of significance, and the size of each dot reflects the proportion of enriched genes within each pathway. This analysis identifies critical pathways that are highly enriched in tumors, providing potential targets for therapeutic intervention. Furthermore, Figure 6H displays a radial plot of GO enrichment analysis.

### Anti-inflammatory and antioxidative effects of DHA and EPA on LPS-stimulated RAW 264.7 cells and cancer cells

**FIGURE 7:**
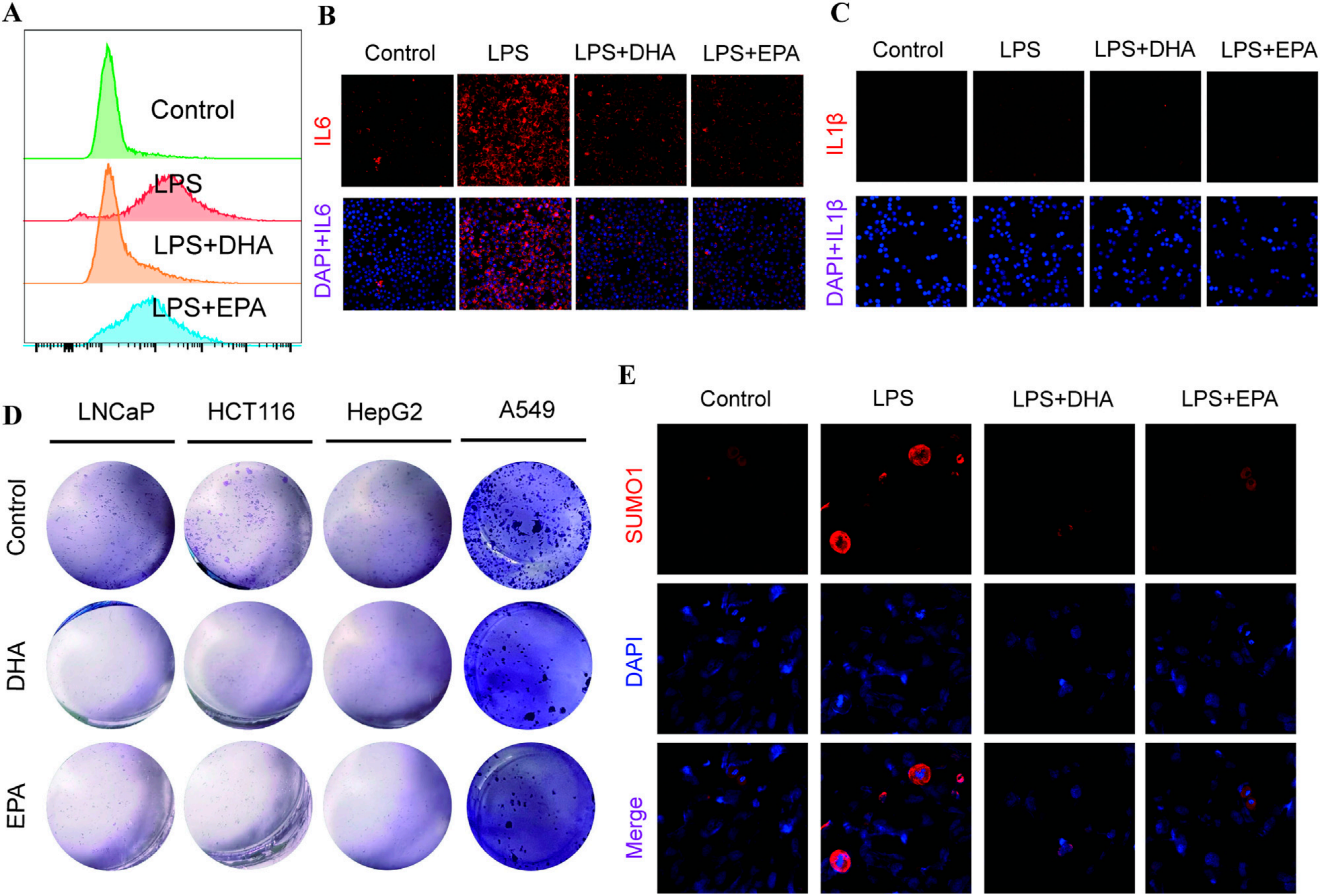
Effects of DHA and EPA on Oxidative Stress, Inflammatory Cytokine Expression, and SUMO1 Nuclear Translocation in RAW 264.7 Cells and Cancer Cell Lines. **(A)** Flow cytometry analysis of reactive oxygen species (ROS) levels in RAW 264.7 cells treated under different conditions: Control, LPS-stimulated (LPS), LPS + DHA, and LPS + EPA. The *x*-axis represents “ROS Intensity (Arbitrary Units),” and the *y*-axis indicates “Cell Count.” DHA and EPA treatments significantly reduced ROS levels in LPS-stimulated cells, demonstrating their antioxidative effects. **(B)** Immunofluorescence staining for IL-6 in RAW 264.7 cells under the following conditions: Control, LPS, LPS + DHA, and LPS + EPA. Both DHA and EPA treatments significantly inhibited LPS-induced IL-6 expression, indicating an anti-inflammatory effect. **(C)** Immunofluorescence staining of IL-1β in RAW 264.7 cells under Control, LPS, LPS + DHA, and LPS + EPA conditions. IL-1β appears in red, with DAPI-stained nuclei in blue. Merged images demonstrate intracellular IL-1β localization. Treatment with DHA and EPA reduced LPS-induced IL-1β expression, further supporting their anti-inflammatory properties. **(D)** Colony formation assay images showing the clonogenic potential of cancer cell lines (LNCaP, HCT116, HepG2, and A549) after treatment with DHA and EPA (300 μM) compared to untreated controls. DHA and EPA treatments resulted in a marked decrease in colony formation across all cell lines, suggesting their potential to inhibit cancer cell proliferation. **(E)** Immunofluorescence analysis of SUMO1 nuclear translocation. This reduction in SUMO1 nuclear translocation suggests that DHA and EPA may modulate post-translational modifications involved in stress response pathways.

This study evaluated the antioxidative and anti-inflammatory effects of docosahexaenoic acid (DHA) and eicosapentaenoic acid (EPA) on LPS-stimulated RAW 264.7 macrophages and various cancer cell lines, demonstrating a marked reduction in oxidative stress, inflammatory cytokine expression, and cancer cell proliferation. Flow cytometry analysis revealed that both DHA and EPA significantly reduced ROS levels in LPS-stimulated RAW 264.7 cells (Figure 7A). Compared to the LPS-only group, cells treated with DHA and EPA exhibited notably lower ROS levels. Immunofluorescence staining was employed to assess the expression of IL-6 and IL-1β in RAW 264.7 cells. In the DHA + LPS and EPA + LPS treatment groups, IL-6 expression was significantly reduced compared to the LPS-only group (Figure 7B). Similarly, DHA and EPA treatments decreased IL-1β expression, a key pro-inflammatory cytokine, as indicated by reduced red fluorescence (Figure 7C). These findings suggest that DHA and EPA effectively suppress LPS-induced inflammatory responses at the cellular level. The effects of DHA and EPA on cancer cell proliferation were evaluated using colony formation assays in various cancer cell lines (LNCaP, HCT116, HepG2, and A549). Compared to the untreated control group, cells treated with DHA and EPA showed a marked reduction in colony formation, indicating a significant inhibitory effect on cancer cell proliferation (Figure 7D). This finding implies that DHA and EPA may possess anticancer properties by inhibiting cell growth. Immunofluorescence analysis also examined the nuclear translocation of SUMO1, a protein involved in stress response pathways, under inflammatory conditions. In the LPS-stimulated group, there was a notable increase in SUMO1 nuclear localization (Figure 7E). Treatment with DHA and EPA resulted in reduced SUMO1 nuclear translocation, suggesting that these fatty acids may modulate stress-related post-translational modifications, further enhancing their anti-inflammatory effects. In conclusion, these findings indicate that DHA and EPA exert antioxidative and anti-inflammatory effects in LPS-stimulated RAW 264.7 cells by reducing ROS production and downregulating pro-inflammatory cytokine expression. Their ability to inhibit cancer cell proliferation and regulate SUMO1-associated nuclear translocation highlights their potential therapeutic applications in managing inflammation and cancer.

## Discussion

In this study, we utilized two-sample Mendelian randomization to investigate the causal relationship between metabolites and PH (Alhathli et al., 2023; Wang et al., 2021). Our analysis identified 57 metabolites that are significantly and causally associated with PH. Notably, piperidine, glucuronide, and N-lactose-valine were positively correlated with PH, while scoparone (C26) and docosahexaenoic acid (DHA) exhibited negative associations with PH (Re et al., 2023). These findings underscore the metabolic basis of PH and suggest pathways that may contribute to its pathogenesis. Additionally, our pan-cancer genomic analysis uncovered substantial alterations in copy numbers, methylation patterns, and TMB of ASOH genes associated with both PH and various malignancies. Key genes, including IL6, IL1B, and COL1A1, emerged from GSEA and tumor prediction analysis, highlighting the broad relevance of these genomic alterations across diseases.

In this study, we strengthen our understanding of the directional relationships between specific metabolites and the pathogenesis of PH, underscoring their substantial impact on modulating PH risk (Astore and Gibson, 2024; Choudhury et al., 2021). The substantial overlap in metabolically regulated genes—such as IL6, IL1B, and COL1A1—in lung epithelial cells associated with PH and all published cancer analyses (where metabolites were elevated) suggests shared mechanisms linking transcriptional changes to genomic alterations (Thakur and Chen, 2019; Ibrahim and Muhammad, 2020). The identification of 57 metabolites causally linked to PH significantly enhances our understanding of its metabolic pathogenesis. Moreover, phenolic compounds found in lentils possess long-lasting antioxidant properties, allowing them to effectively reduce symptoms of PH by decreasing oxidative stress (Xia et al., 2023; Park et al., 2024; Żuchowski et al., 2021). This aligns with studies on the influence of arsenic sulphide on the spread of cancer to other parts of the body through the HIF-1α/VEGF pathway, emphasizing comparable connections between substances produced during metabolism and changes in the genes in pulmonary hypertension (Lu et al., 2023). The study of exercise-induced biomarkers and their relationship with PH offers a new viewpoint on non-pharmacological therapies and their processes (Yau et al., 2016; Reis, 2020). Our research elucidates the roles of DHA and EPA in regulating ROS production and inflammatory cytokine expression, both of which are pivotal in improving endothelial function and mitigating vascular inflammation associated with PH.

The presence of metabolites such as the glucuronide of piperidine and N-lactoyl-valine demonstrates a positive association with the progression of PH pathophysiology, suggesting their potential role in exacerbating the condition. Conversely, the negative associations with metabolites such as scoparone and DHA suggest that these compounds may function as protective agents and hold potential as therapeutic targets (Li and Zhao, 2020; Chen et al., 2020). These findings are consistent with previous research emphasizing the role of metabolic dysregulation in cardiovascular diseases, while extending this focus specifically to PH (Chan and Rubin, 2017; Harvey and Chan, 2017). Additionally, the pan-cancer analysis provides critical insights into genomic alterations associated with the overlapping genes identified (Gröbner et al., 2018; Murphy et al., 2016; Pleasance et al., 2020). Notably, the observed changes in copy number, methylation patterns, and TMB profiling are interconnected, highlighting their essential role in understanding both cancer development and evolution (Thomson et al., 2017; Chen C. et al., 2022). This supports the hypothesis of a shared inflammatory etiology between PH and various cancer types, particularly through genes such as IL6 and IL1B that are strongly associated with inflammation and immune responses (Naugler and Karin, 2008; Taniguchi and Karin, 2014). Furthermore, collagen genes like COL1A1 and COL5A1 emphasize the involvement of extracellular matrix remodeling in both conditions (Dzobo et al., 2012; Machol et al., 2022). DHA and EPA significantly alleviate vascular inflammation and remodeling, which may be related to the regulation of PPAR and NF-κB pathways, ultimately helping to improve pulmonary vascular function (Łacheta et al., 2019). Some fatty acids such as DHA and EPA can downregulate pro-inflammatory cytokines that promote tumor growth, which may improve the effectiveness of conventional treatments such as chemotherapy and radiotherapy (Silva et al., 2015). In addition, DHA and EPA can serve as adjunctive therapies for cancer treatment. Combining these omega-3 fatty acids can help improve patients’ quality of life by reducing chemotherapy related side effects and enhance overall treatment efficacy by regulating inflammatory responses (Silva et al., 2015). DHA and EPA have the potential to serve as dietary supplements to support immune function and alleviate cachexia, a common complication in cancer patients (Szlendak and Kapała, 2024). This is consistent with several studies suggesting that signaling networks, such as the APC/Wnt/β-catenin pathway, are crucial in cancer, corroborated by our findings on genetic changes occurring in PH (De Jesus Perez et al., 2012; Billmann et al., 2018). These results underscore the importance of a holistic approach for studying and treating this complex disease (Chen et al., 2023; Hiremath et al., 2022; Li et al., 2023), emphasizing the significant impact of the microenvironment on tumor development and progression (Mei et al., 2024; Wan et al., 2024).

While this study is comprehensive, it still have limitations. The utility of MR analysis for causal inference can be affected by the reliability and accessibility of genetic instruments (LaPierre et al., 2023; Slob and Burgess, 2020). The findings may also be complicated by pleiotropy, where genetic variants influence multiple traits. Additionally, biases arising from specific cohorts or datasets can lead to selection bias and limit the generalizability of the results. Conclusions derived from single-cohort-based GWAS data may have restricted applicability. Variability in genetic associations across different populations may lead to confounding. It is crucial to recognize and address limitations inherent in MR research, such as pleiotropy, which can confound the analysis. Employing sensitivity analyses and robust genetic instruments could improve methodological transparency. In addition, measuring exposure such as metabolite levels, may result in attenuation of causal estimates, thereby affecting the strength of associations observed. Despite the pan-cancer study’s breadth, it may not capture all genetic changes due to cancer heterogeneity (Tan et al., 2015; Raynaud et al., 2018; Nakamura et al., 2020). Future research should replicate these findings in larger, more diverse populations to deepen our understanding of the underlying mechanisms (Melamud et al., 2020; Frank et al., 2006).

Our findings resonate with earlier studies that established the significant role of metabolites in cardiovascular diseases and cancer, suggesting that metabolic factors influence disease progression and severity (Ussher et al., 2016; Wang and Zhao, 2018). Our integrative approach, combining MR and pan-cancer analysis, uniquely elucidates the interconnected relationship among metabolites, PH, and genomic perturbations, distinguishing it from prior studies focused on specific diseases (Zhong et al., 2022; Pocza, 2012). This research carries substantial implications for clinical practice and future research directions. Understanding the roles of specific metabolites in PH may facilitate the development of targeted therapeutics aimed at regulating these metabolic pathways (Kaddurah-Daouk et al., 2014). The pan-cancer analysis also identified genomic aberrations that could serve as biomarkers for early cancer detection and prognosis, potentially leading to new therapeutic interventions (Ding et al., 2019; Ibrahim et al., 2022). For instance, network pharmacology and molecular docking studies have explored the mechanisms of white peony medicinal herb in treating lupus nephritis (Cao et al., 2023). Subsequent studies have demonstrated the impact of HER2 on bladder cancer cell properties, revealing its contributory role in bladder cancer progression and clinical outcomes (Li et al., 2024). Additionally, PRMT5 has emerged as a key mediator of AKT’s oncogenic activity through methylation, promoting tumor cell metastasis, and targeting PRMT5 shows promise for cancer therapy (Huang et al., 2022). Thus, this integrative approach is powerful, particularly when considering both metabolic and genetic factors in disease processes (Allum and Grundberg, 2020; Barroso and McCarthy, 2019). By integrating several investigations, a thorough comprehension of the connections between metabolic pathways and cancer genomes is achieved, therefore opening up possibilities for tailored treatments focused on metabolism for PH (Bi et al., 2021; Gómez-Cebrián et al., 2022; Le et al., 2019). By combining state-of-the-art advancements and techniques, such as the application of ultrasound to study non-invasive brain-machine interfacing, there is potential to significantly transform the methods used for diagnosing and treating PH (Jones and Hynynen, 2019). The therapeutic implications of pharmacological drugs and the comprehension of drug mechanisms are crucial (Cracowski et al., 2022). Furthermore, examining the significance of trace elements in the diagnosis of atherosclerosis provides fresh perspectives on potential biomarkers for diagnosis and targets for treatment in pulmonary hypertension (Meng et al., 2023; Prashar et al., 2017; Bargieł et al., 2021).

Our research found that DHA and EPA suppress oxidative stress and inflammatory responses in LPS-stimulated RAW 264.7 cells and cancer cells, reducing nuclear translocation of SUMO proteins commonly associated with inflammation. SUMOylation is a critical protein modification process that typically plays a key role in cellular responses to stress and inflammation (Guo and Henley, 2014). When cells experience oxidative stress or inflammatory stimuli, SUMOylated proteins may translocate to the nucleus to enhance the expression of stress-response-related genes (Pascual et al., 2005). However, if DHA and EPA can mitigate inflammation and oxidative stress, this nuclear translocation activity of inflammation-associated SUMO proteins would decrease accordingly, potentially reducing the persistence and intensity of inflammatory responses. In Alzheimer’s disease research, Chen et al. were the first to reveal the role of SUMOylation in regulating neuroinflammation, particularly through SUMO modification of the insulin-like growth factor 1 receptor (IGF1R) in influencing neuroinflammation (Chen et al., 2024a). Additionally, appropriately controlling the nuclear translocation of CRM1-mediated SUMOylated PKM2 protein can significantly reduce neuroinflammation and improve cognitive function (Chen et al., 2024b). Although this finding requires further investigation, it provides potential insights into developing new neuroprotective strategies. Similarly, SUMOylation can modulate the activity of key transcription factors (such as NF-κB and STAT1) in inflammatory signaling pathways, thereby reducing or enhancing inflammatory responses (Parra-Peralbo et al., 2021). For instance, in murine models, inhibiting NF-κB SUMO modification significantly reduces inflammatory responses, decreasing the release of pro-inflammatory factors like IL-6 and IL-1β (Yang et al., 2021; Decque et al., 2016). This inhibition may achieve anti-inflammatory effects by reducing NF-κB nuclear translocation, providing theoretical support for the anti-inflammatory mechanisms of DHA and EPA. SUMOylation also exhibits an important dual role in cancer, potentially promoting tumor progression or inhibiting it by regulating specific pathways. For example, SUMOylation can regulate the activity of tumor suppressor factors such as p53 and HIF-1α, thereby influencing apoptosis and tumor progression (Lee et al., 2017). Some anticancer drugs, such as SUMO E1 inhibitors like TAK-981, are being investigated to suppress the abnormally increased SUMO activity in cancer cells, thereby inhibiting cancer cell proliferation by affecting the nuclear translocation of tumor-related genes (Kukkula et al., 2021). As anti-inflammatory and antioxidant agents, DHA and EPA may act through similar mechanisms, showing promising applications in suppressing inflammation, enhancing cellular function, or exhibiting anti-tumor properties.

These research findings significantly enhance the clinical understanding of PH and its connection with cancer, advancing the current knowledge of the pathophysiological mechanisms linking these two conditions. On one hand, this study offers crucial insights for developing early diagnostic techniques or biomarkers to detect PH in cancer patients, which could lead to earlier intervention and improved patient management. On the other hand, these findings open up potential therapeutic avenues by identifying novel targets and approaches for treating PH within the cancer context, promising to improve overall patient outcomes. DHA and EPA demonstrate substantial potential in alleviating oxidative stress and inflammation, showcasing therapeutic relevance in clinical settings. This study elucidates the molecular mechanisms through which these fatty acids exert their effects, including clinical trials assessing DHA and EPA’s efficacy in specific cancer types. These trials provide alternative therapeutic options and tailored treatment strategies for patients. Future studies should prioritize validating these findings through experimental and clinical trials to substantiate their therapeutic applications. Further exploration of the pathways connecting metabolites with PH and cancer could reveal novel therapeutic targets. Incorporating multi-omics approaches, such as proteomics and transcriptomics, into pan-cancer studies will provide a more holistic view of disease processes, offering deeper insights into pathogenesis.

## Conclusion

This study sheds new light on the metabolically driven causative linkages in PH, as well as the pan-cancer genomic landscape of overlapping genes. The researchers integrated findings from Mendelian randomization studies and pan-cancer data to identify key factors related to metabolism and genetic changes that may serve as potential treatment targets or biomarkers. By tailoring intervention measures based on individual biomarker characteristics, clinicians can improve treatment outcomes and reduce adverse reactions, thereby improving patient prognosis. Future research could focus on validating identified metabolite biomarkers in well-defined clinical cohorts. To ensure the robustness of the research results, it is recommended to conduct multicenter clinical trials to enhance the generalizability of the efficacy of biomarkers for different populations and cancer types.

## Data Availability

The original contributions presented in the study are included in the article/Supplementary Material, further inquiries can be directed to the corresponding authors.

## Abbreviations

PH, Pulmonary Hypertension; MR, Mendelian Randomization; CNV, Copy Number Variation; TCGA, The Cancer Genome Atlas; TMB, Tumor Mutation Burden; PTM, Post-Translational Modification; ROS, Reactive Oxygen Species; IL, Interleukin; DHA, Docosahexaenoic Acid; EPA, Eicosapentaenoic Acid.
