# Supplementary material for: Metabolic pathways, genomic alterations, and post-translational modifications in pulmonary hypertension and cancer as therapeutic targets and biomarkers
Source: Front Pharmacol. 2024 Nov 20;15:1490892. doi: 10.3389/fphar.2024.1490892 (PMC11614602; doi:10.3389/fphar.2024.1490892)
Supplement: Supplementary file 1 [file DataSheet1.docx]

**Supplementary methods**

**Design of MR Research**

A two-sample Mendelian randomisation (MR) analysis was utilised in our investigation to examine the causal association between PH and 1400 metabolites. Genetic variation is used by MR as a useful variable for risk factors. Three requirements must be met for instrumental variables (IVs) to be considered valid in causal inference: (1) the genetic variant and the exposure must be directly correlated; (2) the genetic variant and the outcome must be free from confounding factors; and (3) the genetic variant and the outcome must only influence each other through the exposure. The R10 FinnGen study, which comprised 289,365 Europeans, provided the data on pulmonary hypertension. Of these, 248 had pulmonary hypertension, while the remaining 289,117 were controls.

**Metabolite GWAS Data Source**

The metabolite summary data used in our investigation are taken from the GWAS catalogue, which is available to the public. Specifically, we used registration numbers GCST90199621 through GCST90201020. This comprehensive GWAS dataset comprises 1,091 metabolites and 309 ratios of metabolites, sourced from a cohort of 8,299 individuals participating in the Canadian Longitudinal Study on Aging (CLSA).

**The Instrumental Variables (IV) Selection Process**

To ensure a robust association between genetic variation and exposure, we established a significance threshold of 1 × 10⁻⁵ for each metabolite when selecting instrumental variables. The "TwoSampleMR" software was employed to identify suitable instrumental variables using a clumping distance of 10,000 kb and a linkage disequilibrium (LD) threshold of R² < 0.001. To align with the standard genome-wide significance level in GWAS, we adjusted the threshold for pulmonary hypertension to 5 × 10⁻⁵. We clumped SNPs based on proximity, applying an LD threshold of R² < 0.001, with a clumping distance of 10,000 kb.

**Statistical Analysis**

Statistical analyses were conducted using R software version 4.2.1 for data management and figure generation (R Development Core Team, 2011). The software is accessible at http://www.Rproject.org. Inverse variance weighting (IVW) and weighted median estimation were two primary methods employed to evaluate the causal relationship between 1,400 metabolites and pulmonary hypertension. The analyses utilized the "TwoSampleMR" package (version 0.5.7) in R, which is specifically designed for Mendelian randomization (MR) studies, encompassing causal effect estimation, instrumental variable analysis, hypothesis testing, and sensitivity analysis. The IVW method, commonly applied in MR, integrates Wald estimates from multiple genetic instruments. Estimates were calculated as the number of SNP-outcome connections divided by the total associations between SNPs and exposures, and these estimates were conditionally weighted based on the inverse variance of each SNP-outcome association. Additionally, the package includes various methods such as weighted median and mode-based analyses to provide robust causal estimates, including those that may be invalid under the strong instrument assumption. Utilizing results from numerous studies, we performed extensive sensitivity analyses, incorporating Cochran's Q-test, to evaluate the relationship between instrument factors and heterogeneity.

**Supplementary Figures**

**
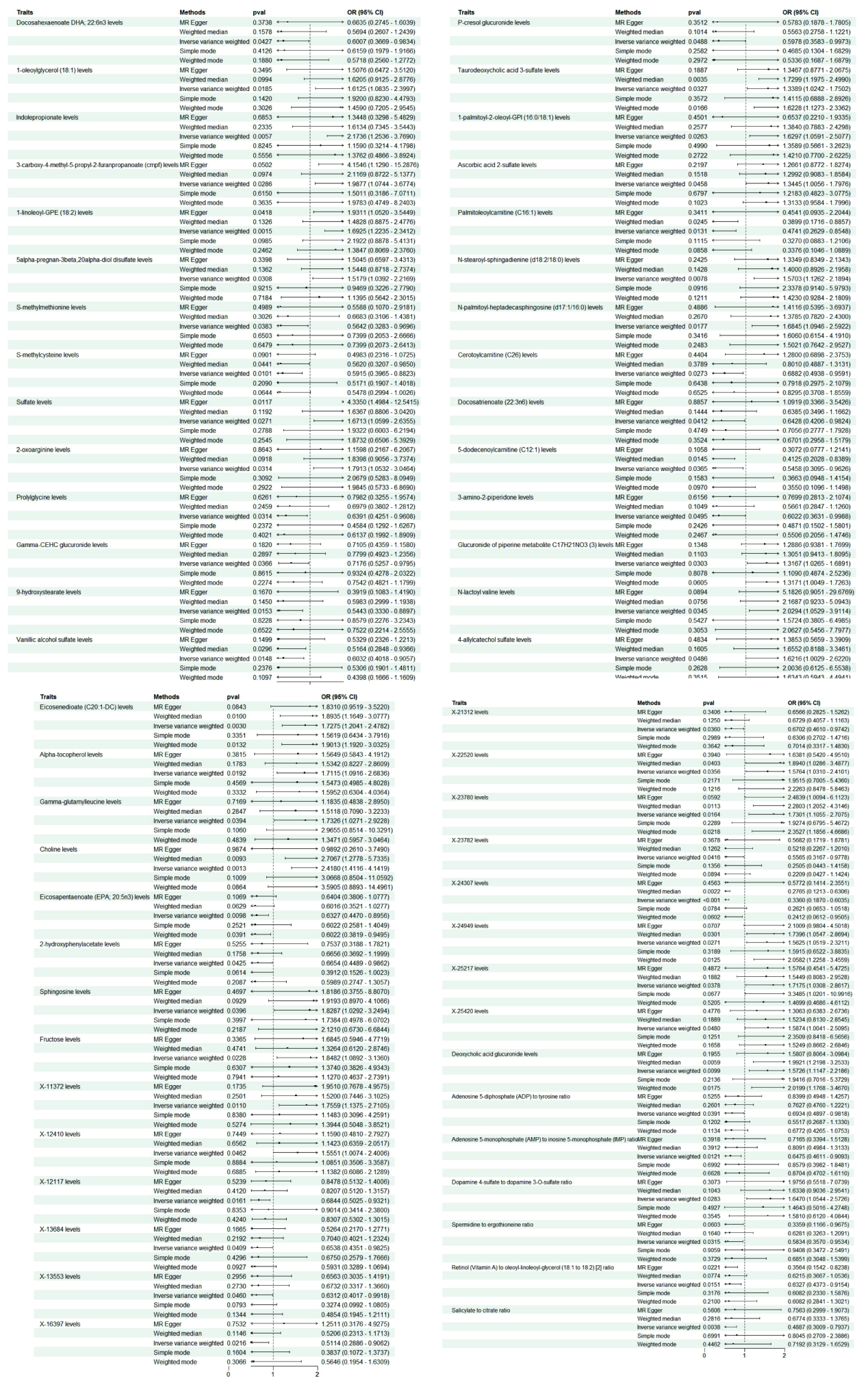
**

**Supplementary Figure 1. MR Forest Plot.**

This figure presents the MR (Mendelian Randomization) forest plots that illustrate the associations between various metabolites and a specific health outcome. Each plot shows the odds ratio (OR) with a 95% confidence interval (CI) for individual metabolites, analyzed using multiple MR approaches, including Inverse Variance Weighted (IVW), MR Egger, Weighted Median, Weighted Mode, and Simple Mode. The metabolites analyzed encompass a range of biological compounds, such as amino acids, lipids, carbohydrates, and other small molecules. In the forest plot, horizontal lines indicate the OR and 95% CI for each metabolite. Lines that intersect the null value (OR = 1) suggest a lack of statistical significance, while lines entirely on one side of the null value indicate a statistically significant effect. The MR analysis leverages extensive genetic datasets, with genetic markers for each metabolite obtained through genome-wide association studies (GWAS). The MR methods used include IVW, which combines effect estimates of genetic instruments for the exposure and outcome; MR Egger, which addresses directional pleiotropy by incorporating an intercept term in the regression model; Weighted Median, which provides a robust estimate even if up to 50% of instruments are invalid; Weighted Mode, which relies on the most common effect among genetic instruments; and Simple Mode, which uses mode-based estimation for causal inference. The central section of the diagram lists the metabolites and their respective codes, along with the p-values for each analytical method, indicating the statistical significance of each association. The rightmost section displays the odds ratio (OR) with a 95% confidence interval (CI) for each metabolite.


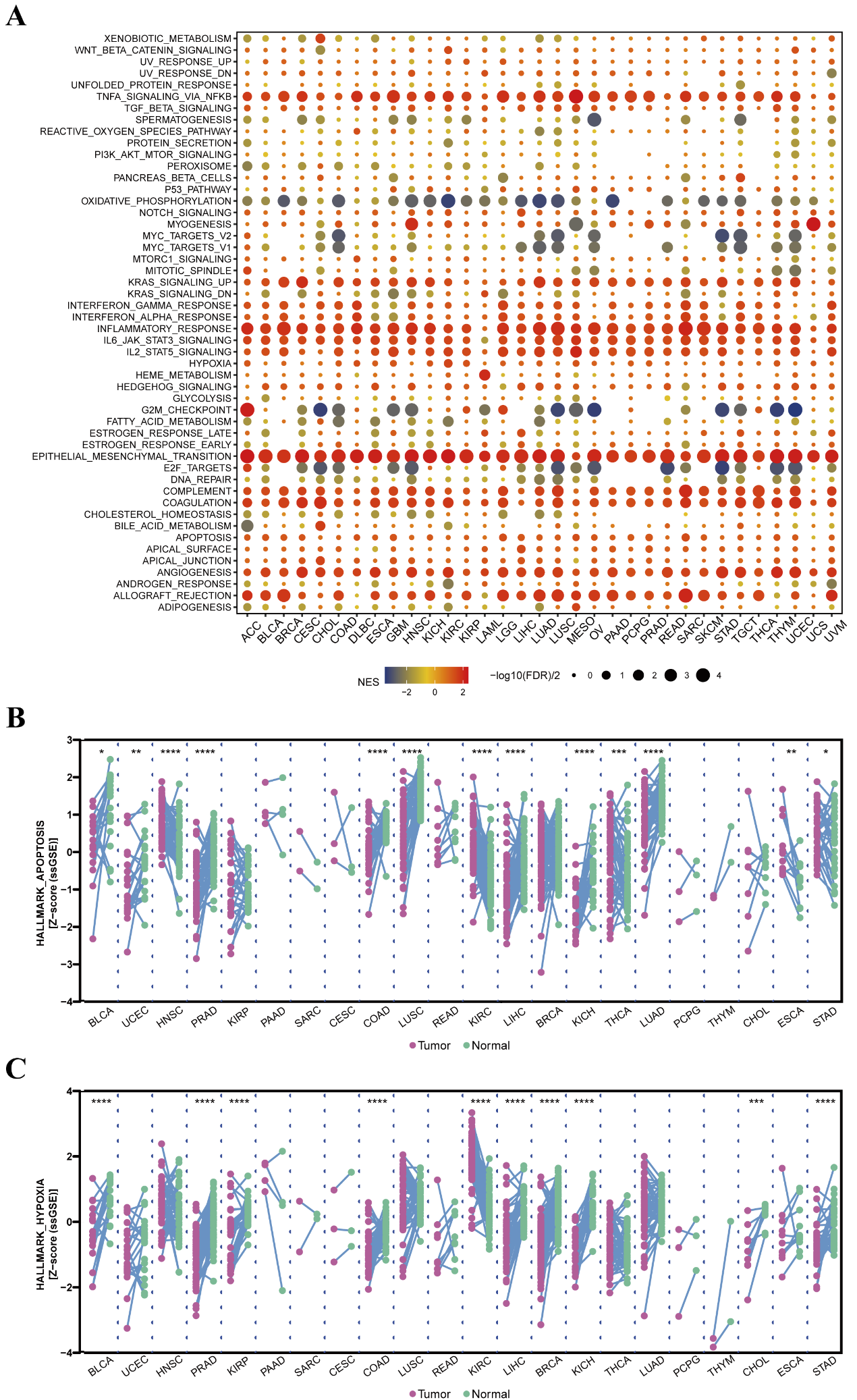


**Supplementary Figure 2. Enrichment Analysis Across Different Cancers.**

GSEA Enrichment Pathways and Scores Across Various Cancer Types

This chart presents the results of Gene Set Enrichment Analysis (GSEA) performed across multiple cancer types. The y-axis lists the pathways analyzed, including xenobiotic metabolism, WNT/β-catenin signaling, unfolded protein response, and epithelial-to-mesenchymal transition. The x-axis displays different cancer types, such as CESC, GBM, HNSC, KIRC, LAML, LIHC, LUAD, LUSC, OV, PAAD, PRAD, READ, SKCM, STAD, TGCT, and UVM. Each dot in the plot represents the normalized enrichment score (NES) for a specific pathway within a particular cancer type. The size of each dot indicates the significance of the enrichment, calculated as -log10 of the FDR q-value divided by 2. The color gradient from blue to red reflects the NES, where blue denotes negative enrichment and red indicates positive enrichment. Across cancer types, notable enrichment is observed in pathways such as WNT/β-catenin signaling, unfolded protein response, interferon-alpha response, and epithelial-to-mesenchymal transition. The analysis was conducted using GSEA software with gene sets from the Molecular Signatures Database (MSigDB), with FDR criteria set at < 0.25 to determine significance.
